# Supplementary material for: Analysis of PPARγ Signaling Activity in Psoriasis
Source: Int J Mol Sci. 2021 Aug 10;22(16):8603. doi: 10.3390/ijms22168603 (PMC8395241; doi:10.3390/ijms22168603)
Supplement: Supplementary file 1 [file ijms-22-08603-s001.zip › Supplemental materials_Analysis of PPARg signaling activity in psoriasis/Pathway models/Models images and html files/Interleukin-17 and interleukin-22 signaling in psoriasis/10231.html]

S100A8


# Protein S100A8

|  |  |
| --- | --- |
| URN | urn:agi-llid:6279 |
| Total Entities | 0 |
| Connectivity | 2034 |
| Name | S100A8 |
| Description | S100 calcium binding protein A8 |
| Notes | The protein encoded by this gene is a member of the S100 family of proteins containing 2 EF-hand calcium-binding motifs. S100 proteins are localized in the cytoplasm and/or nucleus of a wide range of cells, and involved in the regulation of a number of cellular processes such as cell cycle progression and differentiation. S100 genes include at least 13 members which are located as a cluster on chromosome 1q21. This protein may function in the inhibition of casein kinase and as a cytokine. Altered expression of this protein is associated with the disease cystic fibrosis. Multiple transcript variants encoding different isoforms have been found for this gene. [provided by RefSeq, Jan 2016] |
| ObjectType | Protein |

---

|  |  |
| --- | --- |
| Pathway | AHR in Intestinal Cell Antimicrobial Barrier Maintenance |
|  | Proteins Involved in Atherosclerosis |
|  | RAGE/AGER and S100 Proteins in Cardiovascular Injury |
|  | Proteins Involved in Myocarditis |
|  | Proteins Involved in Atopic Dermatitis |
|  | IL22 Induces Keratinocyte Proliferation in Psoriasis |
|  | Proteins with Altered Expression in Psoriasis |
|  | Dendritic Cells Function in Psoriasis |
|  | Advanced Glycation End Products Pathway in Diabetic Microangiopathy |
|  | Proteins Involved in Cystic Fibrosis |
|  | Dendritic Cell Dysfunction in Psoriatic Arthritis |
|  | Proteins Involved in Amyotrophic Lateral Sclerosis |
|  | Immune System Role in Otitis Media |
|  | Proteins Involved in Bronchiectasis |
|  | Proteins Involved in Chronic Obstructive Pulmonary Disease |
|  | Myeloid Derived Suppressor Cells in Cancer Immune Escape |
|  | IFNG/IFNR Expression Targets |
|  | IL10 Expression Targets |
|  | IL22 Expression Targets |
|  | GPCRs Family -> Expression Targets in Lymphoid System and Blood |
|  | Elevated Receptors -> Expression Targets in Bone Marrow |
|  | IL1A Expression Targets |
|  | VEGFA -> STAT Expression Targets |
|  | pprarg neg, uknown targets, ps-positive |
|  | dsRNA induces innate immunity and INFs in alveolar cells |
|  | AGER feed forward loop in innate immunity |
|  | 2\_Interleukin-17 and interleukin-22 signaling in psoriasis |
|  | 3\_Arterial wall inflammation and calcification in atherosclerosis |
|  | drug-target moltransport |
|  | Interleukin-17 and interleukin-22 signaling in psoriasis |

---

|  |  |
| --- | --- |
| MedScan ID | 6279 |

---

|  |  |
| --- | --- |
| LocusLink ID | 6279 |
|  | 20201 |
|  | 116547 |

---

|  |  |
| --- | --- |
| Alias | 60B8AG |
|  | Leukocyte L1 complex light chain |
|  | Cystic fibrosis antigen |
|  | B8Ag |
|  | Migration inhibitory factor-related protein 8 |
|  | MRP8 |
|  | CGLA |
|  | urinary stone protein band A |
|  | chemotactic cytokine CP-10 |
|  | OTTHUMP00000015329 |
|  | L1Ag |
|  | S108\_HUMAN |
|  | CP X |
|  | pro-inflammatory S100 cytokine |
|  | S100 calcium binding protein A8 |
|  | Migration inhibitory factor-related protein VIII |
|  | OTTHUMP00000015330 |
|  | chemotactic s100 protein |
|  | S100a8 |
|  | CP-10 |
|  | AI323541 |
|  | CAGA protein |
|  | S100 calcium binding protein A8 (calgranulin A) |
|  | calprotectin L1L subunit |
|  | CAGA mRNA |
|  | CP10 |
|  | calgranulin A |
|  | CFAG |
|  | MA387 |
|  | P8 |
|  | MIF |
|  | NIF |
|  | CAGA |
|  | protein S100-A8 |
|  | MRP-8 |
|  | S100 calcium-binding protein A8 |
|  | calgranulin-A |
|  | S108 |
|  | S100 calcium-binding protein A8 (calgranulin A) |

---

|  |  |
| --- | --- |
| GO ID | 0050786 |
|  | 0035662 |
|  | 0050544 |
|  | 0005509 |
|  | 0008017 |
|  | 0008270 |
|  | 0006919 |
|  | 0002526 |
|  | 0019730 |
|  | 0006915 |
|  | 0014002 |
|  | 0035425 |
|  | 0006914 |
|  | 0032602 |
|  | 0002544 |
|  | 0001816 |
|  | 0042742 |
|  | 0050832 |
|  | 0006954 |
|  | 0045087 |
|  | 0002523 |
|  | 0070488 |
|  | 0030593 |
|  | 0043312 |
|  | 0018119 |
|  | 0051092 |
|  | 0030307 |
|  | 0050729 |
|  | 2001244 |
|  | 0002793 |
|  | 0051493 |
|  | 0045471 |
|  | 0032496 |
|  | 0010043 |
|  | 0032119 |
|  | 0002224 |
|  | 0042060 |
|  | 0062023 |
|  | 0005856 |
|  | 0005829 |
|  | 0070062 |
|  | 0005576 |
|  | 0005615 |
|  | 0045111 |
|  | 0005634 |
|  | 0005886 |
|  | 0034774 |
|  | 0016209 |
|  | 0046872 |
|  | 0006935 |
|  | 0002376 |
|  | 0050727 |
|  | 0005737 |
|  | 0016020 |
|  | 0098869 |
|  | 0006917 |
|  | 0005515 |

---

|  |  |
| --- | --- |
| KEGG ID | hsa:6279 |
|  | mmu:20201 |
|  | rno:116547 |

---

|  |  |
| --- | --- |
| Organism | Homo sapiens {Organism urn:agi-taxid:9606} |
|  | Mus musculus {Organism urn:agi-taxid:10090} |
|  | Rattus norvegicus {Organism urn:agi-taxid:10116} |
|  | Mus musculus |
|  | Homo sapiens |
|  | Rattus norvegicus |

---

|  |  |
| --- | --- |
| Mouse chromosome position | 3 39.9 cM |
|  | 3 43.6 cM |

---

|  |  |
| --- | --- |
| OMIM ID | 123885 |

---

|  |  |
| --- | --- |
| Rat chromosome position | 2q34 |

---

|  |  |
| --- | --- |
| Hugo ID | 10498 |
|  | HGNC:10498 |

---

|  |  |
| --- | --- |
| Human chromosome position | 1q21.3 |
|  | 1q21 |

---

|  |  |
| --- | --- |
| Swiss-Prot Accession | P05109 |
|  | P05109.1 |
|  | P27005 |
|  | Q53X15 |
|  | P27005.3 |
|  | P50115 |
|  | P50115.3 |
|  | A8K5L3 |
|  | D3DV37 |
|  | Q5SY70 |
|  | Q9UC84 |
|  | Q9UC92 |
|  | Q9UCJ0 |
|  | Q9UCM6 |
|  | P31724 |

---

|  |  |
| --- | --- |
| PIR ID | A31848 |
|  | I56163 |
|  | JN0685 |

---

|  |  |
| --- | --- |
| GenBank ID | NC\_000001 |
|  | NM\_001319198 |
|  | NP\_001306127 |
|  | NM\_001319197 |
|  | NP\_001306126 |
|  | NM\_001319196 |
|  | NP\_001306125 |
|  | NM\_002964 |
|  | NP\_002955 |
|  | NM\_001319201 |
|  | NP\_001306130 |
|  | A12027 |
|  | CAA01001 |
|  | AL591704 |
|  | CH471121 |
|  | EAW53330 |
|  | EAW53331 |
|  | M21005 |
|  | AAA36327 |
|  | AA401538 |
|  | AA903259 |
|  | AI126134 |
|  | AK291328 |
|  | BAF84017 |
|  | BC005928 |
|  | AAH05928 |
|  | BP209293 |
|  | BP226540 |
|  | BT007378 |
|  | AAP36042 |
|  | BU935536 |
|  | CR407674 |
|  | CAG28602 |
|  | X06234 |
|  | CAA29580 |
|  | Y00278 |
|  | CAA68390 |
|  | P05109 |
|  | NC\_000069 |
|  | NM\_013650 |
|  | NP\_038678 |
|  | AC122425 |
|  | CH466547 |
|  | EDL15111 |
|  | L76381 |
|  | AAL40132 |
|  | X87966 |
|  | CAA61204 |
|  | AK157837 |
|  | BAE34219 |
|  | AV007095 |
|  | BC078629 |
|  | AAH78629 |
|  | M83218 |
|  | AAB07229 |
|  | S57123 |
|  | AAB25840 |
|  | Y09039 |
|  | P27005 |
|  | NC\_005101 |
|  | XM\_006232565 |
|  | XP\_006232627 |
|  | NM\_053822 |
|  | NP\_446274 |
|  | AC\_000070 |
|  | AAHX01018654 |
|  | AC097705 |
|  | CH473976 |
|  | EDM00536 |
|  | L18891 |
|  | AAA41637 |
|  | P50115 |
|  | AAHY01029034 |
|  | ABM87366 |
|  | CAI19497 |
|  | XP\_011508163 |
|  | ABBA01049433 |
|  | XM\_005245432 |
|  | ABM84421 |
|  | AABR06018860 |
|  | AC\_000133 |
|  | NC\_018912 |
|  | XP\_005245489 |
|  | AC\_000025 |
|  | AMYH02001902 |
|  | XM\_011509861 |
|  | DQ893495 |
|  | DQ896367 |

---

|  |  |
| --- | --- |
| Swiss-Prot ID | S10A8\_HUMAN |
|  | S10A8\_MOUSE |
|  | S10A8\_RAT |

---

|  |  |
| --- | --- |
| Cell Localization | Secreted |
|  | Cytoplasm |
|  | Cytoskeleton |
|  | Cell membrane |
|  | Cytoplasm, cytoskeleton |

---

|  |  |
| --- | --- |
| Ensembl ID | ENSG00000143546 |
|  | ENSP00000357721.1 |
|  | ENST00000368732.5 |
|  | ENSP00000357722.3 |
|  | ENST00000368733.4 |
|  | ENSMUSG00000056054 |
|  | ENSMUSP00000064385.8 |
|  | ENSMUST00000069927.9 |
|  | ENSRNOG00000011557 |
|  | ENSRNOP00000015473.1 |
|  | ENSRNOT00000015473.3 |
|  | ENSRNOT00000015473 |
|  | ENSMUST00000069927 |
|  | ENSP00000357722 |
|  | ENST00000368733.3 |
|  | ENSMUSP00000064385 |
|  | ENSRNOP00000015473 |
|  | ENST00000368733 |

---

|  |  |
| --- | --- |
| MGI ID | MGI:88244 |
|  | 88244 |

---

|  |  |
| --- | --- |
| RGD ID | 620265 |

---

|  |  |
| --- | --- |
| Unigene ID | Mm.21567 |
|  | Hs.416073 |
|  | Rn.31839 |

---

|  |  |
| --- | --- |
| Homologene ID | 2225 |

---

|  |  |
| --- | --- |
| IPI ID | IPI00230768 |
|  | IPI00007047 |
|  | IPI00231370 |

---
